# Investigation of Neuropsychological Dysfunction Among Recovered Nurses: The COVID-19

**DOI:** 10.7759/cureus.58929

**Published:** 2024-04-24

**Authors:** Mohammad Hossein Salemi, Ahmad Alipour

**Affiliations:** 1 Department of Psychology, University of Tehran, Tehran, IRN; 2 Department of Psychology, Payame Noor University, Tehran, IRN

**Keywords:** covid-19, response inhibition, cognitive flexibility, memory, neuropsychological functions

## Abstract

Objective: This investigation aimed to compare the neuropsychological dysfunctions of coronavirus (COVID-19)-recovered nurses to those of healthy nurses.

Methodology: The present research method was descriptive and causal-comparative, in which the statistical population consisted of nurses with a history of COVID disease and working in the COVID department of public hospitals in Isfahan city. The available method selected 30 nurses with a history of illness and compared them with 30 other nurses from the same hospitals. We collected data using the "go/no go" test, the Wisconsin card sorting test (WCST), and direct and inverted word reading tests. We also analyzed the collected data using multivariate analysis of variance.

Results: The results showed a significant difference between nurses with a history of COVID disease and normal nurses in memory performance, the total error of the Wisconsin card sorting test, and the error of committing and inappropriately inhibiting the go/no go task (P < 0.01). However, there is no significant difference between the two groups in the number of classes, the error of perseveration in the Wisconsin test, or the reaction time of the go/no task (P < 0.05).

Conclusion: Therefore, the present study's results indicate that nurses recovering from COVID-19 perform worse than normal nurses in memory functions, cognitive flexibility, and response inhibition within one to three months of recovery.

## Introduction

The Severe Acute Respiratory Syndrome-CoV-2 (SARS-CoV-2) caused the coronavirus epidemic 2019 (COVID-19) outbreak in December 2019. This pandemic had an immense impact on the worldwide healthcare system and impacted the entire population [1]. COVID-19 is classified by the World Health Organization (WHO) as an "infectious disease" that presents symptoms such as fever, dry cough, and exhaustion [1,2]. It spreads quickly through airborne droplets (respiratory secretions) and indirect contact with contaminated secretions [1,2]. During the severe acute phase of the disease, the most prevalent symptoms are difficulty breathing, abnormalities affecting the blood vessels in the brain, and neurological symptoms such as headaches, dizziness, and impaired senses of taste and smell [3].

Moreover, the virus can induce both pulmonary and systemic inflammation, thereby impacting various organs [3]. Researchers acknowledge COVID-19 as a disease that affects multiple organs and presents with a diverse range of symptoms [4]. In line with the hypothesis that the central nervous system (CNS) might be involved in the initial phases of the infection, neurological manifestations, including headache, dizziness or vertigo, impaired taste and smell, muscle dysfunction, stroke, cerebral hemorrhaging, and encephalopathy, are supported by evidence [3]. Multiple studies have also observed a growing prevalence of various neuropsychiatric and neuropsychological abnormalities, including anxiety, mood disturbances, restlessness, cognitive impairment, and acute disorientation [5-7]. It is worth noting that these symptoms, which involve changes in both the nerves and blood vessels, are more commonly observed in elderly individuals who have a severe illness or numerous underlying health conditions [2].

Moreover, numerous investigations have provided evidence of cognitive impairments in individuals diagnosed with COVID-19, both those with modest symptoms and those experiencing severe forms of the disease necessitating hospitalization in the intensive care unit (ICU) [2,8]. The impact of SARS-CoV-2 infection on cognition may be attributed to the virus's indirect engagement with the central nervous system and the damage caused by the disease to various systems, such as systemic inflammation, immune system dysregulation, or hypoxia [9]. Furthermore, additional supporting data indicates a direct infection of the CNS [2]. According to several studies conducted, 80% of patients exhibited neuropsychological impairments [5-7]. The most often observed deficiencies included visuospatial and executive skills, working memory, abstraction ability, and orientation, as assessed by the Montreal Cognitive Assessment (MoCA) test. These symptoms may persist beyond the initial phase of the COVID-19 infection [5]. More information is needed to find out exactly how the disease affected the cognitive functions of people who had persistent COVID-19 symptoms (especially those who did not need to go to the hospital). There are also unclear effects of post-acute COVID-19 on respiratory function, fatigue, and cognitive function because it has only been a short time since the pandemic started [10]. Therefore, this study was conducted to compare neuropsychological dysfunctions in patients who recovered from coronavirus and ordinary people.

## Materials and methods

The present research method was descriptive and causal-comparative, in which the statistical population consisted of nurses working in the COVID department of Isfahan hospitals in the summer of 2022 who had a history of contracting the coronavirus under reference to IIH/1008/2022. Thirty nurses who had a history of COVID disease and whose recovery period was at least one month and at most three months were selected from among the mentioned community as a sample by the available method, and they were compared with 30 nurses who did not have a history of contracting the coronavirus. The inclusion criteria were: informed consent to participate in the research; receiving a diagnosis of COVID by laboratory and paraclinical methods such as C-reactive protein test (CRP) and computed tomography (CT) scan in the last 2 to 5 months; recovering from the disease for at least one month and at most three months; not suffering from acute mental and neurological disorders at the time of study; and being at least 20 and at most 50 years old. The following tools were also used to collect information:

Go/no-go task

This tool measures response inhibition. The original version of the go/no-go task was designed by Hofman in 1984, cited by Wodka et al. [11], and is widely used to assess response inhibition. Response inhibition is executive control over pre-prepared motor responses in response to a change in situation request. This test includes two categories of congruent and incongruent stimuli. The subject must respond to congruent stimuli and avoid responding to incongruent stimuli. The inability to inhibit the response or the error of committing in this test occurs with the motor response when the non-target stimulus is presented. During the process of providing stimuli, the number of congruent or go stimuli is more significant than the number of incongruent or no-go stimuli. The computer runs and scores this test, yielding three distinct scores: the percentage of errors committed, the percentage of inappropriate inhibition, and the reaction time. A high score for committing an error, inappropriate inhibition, and reaction time indicates a person's weakness and inability to inhibit the response. Scoring is done so that a positive score is given for each response consistent with the stimulus, and a negative score is considered for an incongruent response. A negative score is also considered for each omitted answer. The computer also measures the reaction time in milliseconds.

Wisconsin card sorting test

The Wisconsin Card Sorting Test (WCST), used in this study to measure cognitive flexibility, is a neuropsychological test designed by Grant and Berg in 1948 [12,13]. This test is widely used to measure high cognitive processes and brain levels. The Wisconsin Card Sorting Test is specifically considered a measure of the prefrontal cortex and dorsolateral prefrontal cortex function. The 64 cards in this test consist of four types of cards, each of which is different in terms of color (red, blue, yellow, or green), shape (circle, star, triangle, and cross), and number (one, two, three, and four). Each card is assigned one of the four colors and is adorned with one of the four designs: stars, triangles, circles, or crosses. The number of shapes on each card also varies from one to four, so none of the works are identical. This test has different scoring methods. In the most common scoring method, three scores are obtained in this test: perseveration error, total error, and the number of classes. A high score in the first two items indicates the poor performance of the subject, while a high score in the number of classes indicates the excellent performance of the subject. There are several ways to score this test. The most common scoring method is to record the number of classes obtained, perseveration errors, and specific errors. The obtained classes refer to the number of correct periods, or, in other words, ten consecutive correct placements, in the range of zero to six, in which case the test is naturally stopped. There is a perseveration error when the subject continues to categorize according to the previous successful principle and insists on categorizing based on an initial wrong guess in the first series. Notable errors include errors other than perseveration errors, which, together with perseveration errors, form the total error.

Direct and inverted span test

The present study used the direct and inverted word span tests to measure memory. This test was designed by Marconi in 1994 [14]. In this test, you will encounter a test with eight rows of words, in which the words gradually increase with the increase of letters and the syllables of the words. People widely use this test to assess their working memory. The experimenter reads a series of random words, and the subject must repeat the words in the same order. The rows of words initially have two words and eventually reach seven words. When a person repeats an incorrect chain twice in a row, the test stops. The test does not provide any feedback. We score the subject's performance based on the number of correctly recalled series. The inverted pronunciation test follows the same method as the direct word pronunciation test but requires the subject to remember the words in reverse order.

The research was conducted using a procedure that includes sampling and collecting data. The subjects were provided with a concise explanation of the research's goal, and their informed consent was obtained. The researcher evaluated each patient and control group subject individually at the hospital using research tools and provided them with the necessary instructions and explanations to respond to these tools.

## Results

In the present study, 30 patients who recovered from COVID with an average and standard deviation of age of 56.32±5.09 and 30 normal nurses with an average and standard deviation of age of 30.83±4.72 participated. In the patient group, 20 people (66.7%) had a bachelor's education, and 10 (33.3%) had a master's education. In the control group, 21 people (70%) had a bachelor's education, and nine (30%) had a master's education. In addition, in the patient group, seven people (23.3%) had a low socioeconomic status, 16 people (53.3%) had an average socioeconomic status, and seven people (23.3%) had a high socioeconomic status. In the control group, eight people (26.7%), 12 people (40%), and 10 people (33.3%) had low, medium, and high socioeconomic status, respectively (Table 1).

**Table 1:** Demographic information

Parameters	Patient group N (%)	Control group N (%)
Age (in years)	56.32 ± 5.09	30.83 ± 4.72
Level of education		
Bachelors	20 (66.7%)	21 (70%)
Masters	10 (33.3%)	9 (30%)
Socio-economic status		
High	7 (23.3%)	10 (33.3%)
Average	16 (53.3%)	12 (40%)
Low	7 (23.3%)	8 (26.7%)

We used the Mbox test, which examines the homogeneity of variance/covariance matrices, and Levin's test, which examines the homogeneity of variances between groups, before using the parametric test of multivariate analysis of variance to comply with the test's assumptions. None of these tests were significant (P < 0.05); therefore, the condition of homogeneity of variance/covariance matrices was met. Also, as the results of Table 2 show, all three statistical indicators of the test criteria are significant at the level of P < 0.001 regarding the difference between the two groups of patients and normal. This finding means that the two studied groups have a significant difference, at least in terms of one of the dependent variables. The eta squared values obtained by choosing the effect size estimation are proof of the share of the variance that is related to the combined variable of the group and show that the difference between the two groups, according to the dependent variables, is significant in total. The amount of this difference is 50.6%; that is, 50.6% of the variance is related to the difference between the two groups in the mutual influence of the dependent variables.

**Table 2:** Results of significance tests of multivariate analysis of variance for the main effect of group variable on dependent variables F: ANOVA

Name of test	Amount	F	p-value
Pillai’s effect	0.506	7.60	0.001
Lambda effect	0.494	7.60	0.001
Hotelling’s effect	1.02	7.60	0.001

Table 3 shows that there is a significant difference between the two groups in memory, total error (from the components of the Wisconsin card sorting test), and the error of committing and inappropriate inhibition (from the components of the go/no-go test) (P < 0.01). We can conclude that patients who have recovered from COVID-19 perform lower than the regular control group in the neuropsychological functions of memory, cognitive flexibility, and response inhibition, given their lower memory scores and higher scores in total error and inappropriate inhibition. However, no significant difference was observed between the two groups in the number of classes, perseveration error (from the components of the Wisconsin Card Sorting Test), or reaction time (from the components of the go/no-go test) (P < 0.05).

**Table 3:** Results of multivariate analysis of variance of memory scores, cognitive flexibility, and response inhibition in patients recovered from COVID and normal patients SD: standard deviation

Variables	Patient group mean ± SD	Normal group mean ± SD	ANOVA F	p-value
Memory	6.96 ± 1.40	10 ± 3.53	19.10	0.001
Number of floors	2.10 ± 0.99	2.60 ± 1.03	3.75	0.057
Error of preservation	25.03 ± 13.87	30.23 ± 7.25	3.31	0.074
Total error	75.40 ± 9.99	56.20 ± 22.56	18.15	0.001
Committed error	29.47 ± 84.18	23.24 ± 14.43	30.80	0.001
Improper inhibition	78.42 ± 34.24	9.28 ± 31.17	25.7	0.009
Reaction time	0.47 ± 3.0	48 ± 3	2.00	0.967

## Discussion

The present study was conducted to compare the neuropsychological dysfunctions of memory, cognitive flexibility, and response inhibition in patients who recovered from COVID with those of normal nurses. The results showed that there is a significant difference between the two groups in memory and the total error component (from the components of the Wisconsin Card Sorting Test) and the error of committing an inappropriate inhibition (from the components of the go/no-go test). However, there was no significant difference between the two groups in terms of number of classes, perseveration error (from the components of the Wisconsin card sorting test), or reaction time (from the components of the go/no-go test).

These results suggest that patients who recovered from COVID-19 perform less well in memory and response inhibition and, to some extent, in cognitive flexibility. This finding is in agreement with previous research, such as the findings of Tavares-Júnior et al. [15], Alemanno et al. [5], Becker et al. [16], Wu et al. [17], and Zhou et al. [18], based on the presence of neurological and cognitive defects in patients with COVID and recovered from COVID. In the study of Delgado-Alonso et al. [19], results showed individuals afflicted with COVID-19 who have reported cognitive manifestations have exhibited diminished cognitive functioning, particularly in domains associated with attention concentration and executive abilities, episodic memory, as well as visuospatial processing. Conversely, the study by Costas-Carrera et al. [20] revealed a correlation between cognitive impairment and factors like delirium, mechanical ventilation, and inflammation in the ICU. Additionally, cognitive reserve (CR) played a role in mitigating the influence of these variables on cognitive function.

Interestingly, although cognitive complaints were associated with anxiety, there was no significant correlation between cognitive performance and these complaints. A study by Hampshire et al. [21] examined signs of pre-existing intelligence and found that there was no evidence to support the existence of these differences before the infection. However, a more detailed analysis of performance on individual sub-tests provided further support for the notion that COVID-19 exerts a comprehensive influence on various domains of human cognition.

Coronavirus infections can impact the nervous system, which in turn can influence a person's cognition. It is now believed that this virus can transform an initial infection into a continuous infection, leading to neurological and cognitive diseases by coordinating the immune system of the individual. A study on neurological symptoms in infected patients revealed that about 36.4% of COVID-19 patients only display various neurological symptoms [22]. Severe cases exhibit these symptoms more frequently than mild and moderate cases. Also, autopsy reports of this category of patients indicate brain tissue inflammation and partial nerve damage in deceased patients [19]. Besides the previously mentioned lesions, reports have also noted cases of viral brain inflammation (encephalitis) resulting from COVID-19's attack on the central nervous system. Laboratory studies of cerebrospinal fluid have also shown that this virus has the potential to cause severe damage to the nervous system [23]. Therefore, according to the direct relationship between the central nervous system, especially the brain, and cognitive functions, the presence of mental and neuropsychological defects in patients suffering from COVID seems quite clear and logical [24].

Many viral infections can cause severe damage to the structure and function of nerves. Among these injuries, viral infections can cause acute brain inflammation (encephalitis), leading to severe systemic involvement and acute lesions in the myelin of neurons [24].

COVID-19, with a diameter of approximately 100 nm, is spherical or oval and has large spots of glycoprotein on the virus membrane, which also has a single-stranded RNA in its core [25]. The most common and important type of SARS virus can potentially damage the nervous system [26], primarily manifesting in the early stages of the disease [22]. The body's immune system, as a mediator, can intensify this damage because the contamination and destruction of macrophages, microglia, and astrocytes in the central nervous system are of particular importance [24].

In general, it can be said that infections cause the death and destruction of nerve cells in several ways: by disrupting the blood supply to the neurons, disrupting the metabolism of glucose or oxygen in the brain, changing the membrane of nerve cells and the electrical properties of neurons, by producing cerebrospinal fluid that consists of white blood cells and increases the internal pressure of the brain, or by changing the fluid surrounding the nerve cell, they disrupt the function of neurons, and this disruption in the function of neurons also causes disturbances and defects in neuropsychological functions [27].

Limitations of the study

The sample size is relatively limited, consisting of only 30 nurses in each group. Moreover, the inclusion of nurses from the same hospitals may introduce bias, as their exposure to similar environmental conditions could potentially influence cognitive performance. The study exclusively included nurses employed in public hospitals in Isfahan city, hence potentially constraining the applicability of the results to nurses working in different areas or healthcare settings. Assessing neuropsychological dysfunctions between one and three months of recovery may not fully reflect the complete range of cognitive alterations associated with COVID-19. Conducting longitudinal investigations with extended periods of follow-up might yield a more thorough comprehension of cognitive recuperation after contracting COVID-19.

## Conclusions

In conclusion, results indicate that nurses who have recovered from COVID-19 may experience impairments in memory skills, cognitive flexibility, and response inhibition as compared to healthy nurses within a period of one to three months after recovery. Considering the impact of the coronavirus on the neuropsychological functions of both infected and recovered patients, we suggest using cognitive rehabilitation to improve neuropsychological dysfunctions in cases where the severity of dysfunctions is more significant and results in a decrease in performance. Additional studies using larger sample sizes, long-term follow-up, and careful examination of potential confounding variables are necessary to confirm and expand upon these findings.
